# Words matter: towards a new lexicon for ‘nontechnical skills’ training

**DOI:** 10.1186/s41077-019-0098-5

**Published:** 2019-04-27

**Authors:** Paul Murphy, Debra Nestel, Gerard J. Gormley

**Affiliations:** 1 Drama Studies, School of Arts, English and Languages, Queen’s University Belfast, Belfast, UK; 2 Centre for Medical Education, Queen’s University Belfast, Belfast, UK; 3 Simulation Education in Healthcare, Monash University and Department of Surgery, University of Melbourne, Melbourne, Australia

Outsiders to the field of health professions education (HPE) may be intrigued by the phrase ‘nontechnical skills’ (NTS), particularly regarding its usage in the training of healthcare professionals (HCPs). In 2011, Nestel et al. argued that ‘the term NTS is misleading, inaccurate, and oversimplifies critical aspects of professional clinical practice’ [1]. The authors continue, ‘We hope that it is not too late to reframe this thinking. We may need to conceive of ‘skills’ in a different way to avoid reduction in two broad categories’ [1]. In an accompanying editorial, Gaba offers a critique of the argument and states that ‘I do not believe that the answer to this problem is to modify terminology. That would probably be a losing battle anyway. The terms skills and nontechnical skills are both well entrenched in the literature and in common parlance’ [2]. Nonetheless, if simulation-based education (SBE) is supposed to approximate realistic situations, then we need terminology that accurately describes the skills taught in SBE. Words matter because they frame our understanding of the world around us and how we interact with each other in real life. The philosopher Martin Heidegger states that language is ‘the house of being. In its home human beings dwell. Those who think and those who create with words are the guardians of this home’ [3]. In this editorial, we contend that terms such as ‘nontechnical’ and ‘nonverbal’ are inaccurate and that they must be changed as part of the move towards a new lexicon for HPE. Firstly, we examine how the use of the word ‘technical’ has evolved and how the technologization of HPE has resulted in problematic terminology that should be modified. Secondly, we look at the similarities between SBE and actor training and suggest that ‘behavioural’ is a more accurate term than ‘nontechnical’ when describing the range and complexity of interpersonal skills. Thirdly, we make the case for revised HPE curricula where ‘behavioural’ skills can gradually be brought up to par with clinical skills. We conclude by suggesting that a change in language will lead to a shift in attitudes and ultimately a change in educational focus regarding key skills that are often undervalued in HPE.

## Technical skills, language and meaning in a technologized society

As the definition and common understanding of the term ‘technical’ is central to the debate, it is instructive to remember that the word evolved from the ancient Greek word τέχνη, meaning art or craft [4]. So, from the time of Aristotle onwards, the word ‘technical’ implied artistic and creative ability, such as making pottery, building ships for the Athenian navy, winning an event at the Olympics or achieving acclaim in the theatre [5]. As the semantic applications of the term developed in response to changing social and economic requirements, the term ‘technical’ was used to describe practices that were more mechanistic and specific to the prevailing scientific paradigm [6]. The Industrial Revolution led to the increased mechanization of society in the western world and then globally throughout the twentieth century [7]. The Digital Revolution of the late twentieth century further consolidated the integration of sophisticated technology into our daily lives. During this transformation, the terms ‘technical’ and ‘technology’ became associated with electro-mechanical devices including computers, robots and a myriad of gadgets. Broadly speaking, the technologization of society resulted in ‘technical’ skill becoming analogous with using technology as part or instead of the interaction with other people. A technician, for example, is someone who is generally understood to be highly proficient in the use of technology rather than being proficient in terms of interaction with other people. The latter is taken as given insofar as people, for the most part, learn interpersonal skills as a matter of lived experience rather than formal training.

Along with the rest of society, HPE has been transformed by technologization, and increasingly sophisticated electronic devices and manikins have become a common feature of SBE. In terms of the historical development and the changing culture of HPE, the integration of technology involved the implicit adoption of terminology and related attitudes. These were gradually accepted and normalized, resulting in a prioritization of skills involving interaction with technology over skills involving interaction with people. ‘Hard’ science, for example, is prioritized over ‘soft’ skills that students are expected to pick up in the course of everyday life. Governments and related funding bodies prioritize STEM subjects (Science, Technology, Engineering and Medicine) over the Arts, which are perceived as a luxury rather than a necessity [8]. The study of the human body using quantitative methods is prioritized over the study of the human being in a more holistic manner using qualitative methods. The technologized culture has led to a utilitarian approach to language and people typified in the phrase ‘we used standardized patients’. The term ‘used’ objectifies people who perform as patients in SBE. The term ‘engaged’ is better because it reflects the fact that people who perform as patients in SBE are active participants who, in addition to performing a prescribed role in line with the specific requirements of the simulation, can also offer feedback in the debriefing session afterwards [9]. ‘Standardized’ is a word best applied to a product or process that can be calibrated like a machine. ‘Simulated participants’ is a better phrase since it foregrounds the fact of simulation as an attempt to approximate real life rather than a standardized version with little fidelity to the inherent complexity and randomness of human behaviour.

Communications skills are increasingly relevant in HPE, and so it is important to remember the approach advocated by Aristotle’s contemporary, Hippocrates, chiefly that medicine is as much an art as it is a science. Therefore, to describe practices that are not scientific or clinical as ‘nontechnical’ is to relegate them to subordinate status. From the perspective of students in HPE, who must carefully manage their time and energy as they complete a full and complex programme of study, any constituent focus that is not deemed to be essential is, of necessity, deprioritized. We argue that a more balanced approach is needed to reaccentuate focus on techniques used in the interaction between people which, for the sake of brevity, we will call ‘behavioural’ skills.

## Similarities between SBE and actor training

We have found that there are striking similarities between SBE and actor training. We suggest that the combination of these two modes of training offers transformative possibilities for each mode. Moreover, this combination can lead to a new hybrid model of training when the two modes are combined into a new framework in which ‘behavioural’ skills can be taught. Students who engage respectively in actor training and HPE learn to perform technically complex roles in a variety of different settings. The emphasis in HPE has traditionally been on clinical technique over and above ‘behavioural’ technique, which has been relegated to subordinate status with the prefix ‘non’ before words such as ‘technical’ and ‘verbal’.

Konstantin Stanislavski, a Russian actor and director widely regarded as the pioneer of modern actor training, engaged in the most comprehensive study of acting in the modern era that resulted in a synthesis of physical and psychological techniques into a ‘psycho-physical’ approach [10]. The approach was refined over decades of research into an holistic methodology that can enable an actor to create and perform a role that is highly realistic. The level of realism can encourage an audience to willingly suspend their disbelief that a character is merely an aesthetic construct and accept for the duration of the play that the character is a real, live human being. The approach became known as the ‘System’ (a precursor to the ‘Method’ approach developed by Lee Strasberg in the USA) and involves rigorous physical training. The System also involves techniques that tap into emotional memories and harnesses the creativity of the imagination that can enable an actor to embody a character other than himself/herself. The actor defines their role in relation to the ‘super-task’, or governing theme of the play, and breaks their role into manageable sections or ‘episodes’, that help shape basic actions to be carried out. These episodes are in turn broken into smaller ‘tasks’ that can be as small as a facial gesture or a single phrase, but when linked together in the ‘through line’ of action result in a fully realized character that works in concert with the other characters in the play. The level of preparation for performing a role can be exquisitely detailed and refined over weeks of rehearsal. The approach combines high levels of cognition and physical activity when an actor performs a complex role, such as William Shakespeare’s Hamlet or Henrik Ibsen’s Hedda Gabler, in front of a live audience. The actor must immerse himself/herself in the psychological and physical embodiment of a character while simultaneously maintaining sufficient detachment and awareness so that they do not lose themselves in the role.

Actor training, therefore, involves a systematic, methodical and task-driven approach to achieving individualized objectives as part of a team effort to achieve a larger, shared objective that parallels how students are taught in HPE. The detailed approach to learning how to perform a role can offer a coherent framework in which ‘behavioural’ skills can be learned. In terms of ‘technical’ skill, if we were to tell a graduate of a leading acting school that their skills were ‘nontechnical’, their likely response would range somewhere on the spectrum from bemusement to indignation. An actor trained to a high calibre has developed the capability to engage in live performance to such a level of ‘technical’ competency that it is considered an art form. The argument could be made that high a level of competence in acting or ‘behavioural’ skills is not required of HCPs in SBE. We could, however, make the counterpoint that such competence is exactly what should be expected and required, given the significance of the impact of those skills on patient outcomes. To the response that there is not enough space in already packed curricula, the answer is that curricula must be adapted so that ‘behavioural’ skills are given the same priority as clinical skills in SBE.

## Behavioural skills in a revised curriculum

While ‘behavioural’ is a relatively crude term to describe the quality and range of activities encompassed within the category of interpersonal interaction, it is nonetheless preferable to ‘nontechnical’. ‘Behavioural’ encompasses those practices variously described as soft skills, bedside manner, interpersonal, teamwork, leadership and communications to name but a few. In all these aspects, the HCP performs a range of actions following a specific role, be that of the doctor, nurse, midwife, pharmacist or social worker. The impact on the health and wellbeing of patients is at the heart of the encounter. Clinical skills are given the attention they deserve in HPE, but ‘behavioural’ skills less so and must be given the same priority. In the encounter between patients and HCPs, patients are intensely focussed on the voice and movement of HCPs and notice minute expressive details of which HCPs may not be aware. The impact of a facial gesture be that a smile, a frown, a look of disdain, or the raising of an eyebrow can make an immediate and potentially permanent impression on the emotional memory of patients [11]. Furthermore, this impact is also evident between HCPs as they interact with each other. Given the significance of such gestures, the phrase ‘nonverbal’ communication should be changed to ‘physical’ communication in order to place it on a par with verbal communication.

Much has been written about verbal communication in HPE, but the field of ‘physical’ communication or body language as it is more commonly described, can nonetheless be significantly refined. Proxemics or the study of interpersonal space [12] is central to both actor training and stage management [13] and can provide valuable insights for SBE where the focus is on the interaction between HCPs and patients. Presence or the sense of being ‘in the moment’ is another key element of acting and is similarly critical to HCP’s intellectual and emotional states during their encounter with patients. The demeanour of HCPs towards patients can be enhanced by actor training in terms of posture and deportment which will lead to better quality of engagement and better outcomes for patients [14]. Many key skills that actors learn during their training are transferable to the working practice of HCPs in terms of their encounters with colleagues and with patients. We envisage enhanced curricula in HPE in which acting or ‘behavioural’ skills are integrated that provide graduates with enhanced skills and a heightened awareness of interpersonal engagement.

One way to begin this transformation and highlight the importance of ‘behavioural’ skills in HPE is to incorporate modified terms as suggested above. It is important to begin this process early at undergraduate level so that students are immersed in a renewed culture and adopt the terms as given. Apropos of the broader scope of HPE in a global context, this will, of course, involve an incremental process in which the change in language will lead to a change in attitudes and, ultimately, a change in educational focus. This will not happen overnight. Fundamental change takes time. In *Translations* the playwright Brian Friel cautions us to ‘remember that words are signals, counters. They are not immortal. And it can happen […] that a civilisation can be imprisoned in a linguistic contour that no longer matches the landscape of … fact’ [15]. If we can understand ‘civilization’ analogously as the culture of practices in SBE and HPE more widely, then we must ensure that our language matches the landscape of the real world that we are trying to simulate.

## Abbreviations

HCP: Healthcare professional
HPE: Health professions education
NTS: Nontechnical skills
SBE: Simulation-based education
STEM: Science, Technology, Engineering and Medicine.
